# An application of the One Safe Act framework: Proactive safety behaviors in obstetric care

**DOI:** 10.1002/pmf2.70271

**Published:** 2026-03-16

**Authors:** Rebecca M. Cohen, Kimberly B. Glazer, Peter S. Bernstein

**Affiliations:** ^1^ Department of Medical Education Icahn School of Medicine at Mount Sinai New York New York USA; ^2^ Department of Obstetrics and Gynecology University of Pennsylvania Perelman School of Medicine Philadelphia Pennsylvania USA; ^3^ Department of Biostatistics Epidemiology and Informatics University of Pennsylvania Perelman School of Medicine Philadelphia Pennsylvania USA; ^4^ Department of Obstetrics Gynecology and Reproductive Science Icahn School of Medicine at Mount Sinai New York New York USA

**Keywords:** patient safety, proactive safety behaviors, quality improvement

## Abstract

**Objective:**

To perform a qualitative analysis of staff‐reported proactive safety behaviors performed on the obstetric service.

**Study design:**

This qualitative thematic analysis included a convenience sample of obstetric staff at a single tertiary care center who completed the One Safe Act survey, first introduced at Mount Sinai's Quality and Safety Summit (January 2024) and later distributed to all obstetric staff. All obstetric staff were eligible for inclusion. Participants were asked to reflect on their One Safe Act (proactive safety behavior) and record their experience as free text in an online survey tool. We used a combined deductive and inductive analytic approach to identify key themes and categorize self‐reported staff safety behaviors within the obstetric environment.

**Results:**

A total of 141 obstetric staff members participated in the survey. Participants included attendings, fellows, residents, physician assistants, midwives, nurses, and anesthesiologists. A total of seven behavioral themes were identified, ordered from most to least common: (1) *Patient communication, education, and engagement* (26.2%); (2) *Staff communication and team routines* (22.0%); (3) *Protocol adherence* (13.1%); (4) *Personal or team readiness* (13.1%); (5) *Prepared environment, equipment, and resources* (8.9%); (6) *Positive team culture* (8.9%); and (7) *Routine‐based clinical care and documentation* (7.7%).

**Conclusion:**

The One Safe Act survey captured proactive safety behaviors performed by obstetric staff, from which seven themes were identified. This set of themes may serve as a foundation for individual practices and institutional initiatives that promote patient safety.

## 1 INTRODUCTION

The obstetric environment is fast‐paced and inherently unpredictable, often requiring rapid clinical decisions that increase the risk of preventable harm. Patient safety initiatives are especially critical in this setting given the ongoing maternal morbidity and mortality crisis in the United States [1, 2, 3, 4]. For example, in 2020, state maternal mortality review committees determined that over 80% of maternal deaths were preventable [4, 5, 6, 7]. Thus, quality improvement efforts aimed at reducing preventable harm remain essential to enhance patient safety in obstetrics and across healthcare settings.

The One Safe Act tool was developed by Duffy et al. to capture and highlight proactive safety behaviors among perioperative staff [8]. The One Safe Act framework is grounded in the Safety‐II paradigm, which focuses on understanding “what goes right” in complex systems rather than identifying “what goes wrong,” as emphasized in the Safety‐I framework [8, 9]. This approach distinguishes between “work as imagined” and “work as done” [8]. Whereas first‐generation quality assessments, such as root cause analyses or adverse event reviews, typically evaluate errors after they occur, the Safety‐II framework emphasizes a proactive and vision‐based mindset. Furthermore, the One Safe Act tool was designed to contribute to situated learning among staff, in which they can learn through participation and collaboration with colleagues in their community of practice [8, 10].

By developing and administrating the One Safe Act tool, Duffy et al. identified procedure‐specific behaviors such as locking all stretchers in the preoperative area as well as more general behaviors such as verifying patient information [8]. While their study provided valuable insights into proactive safety behaviors utilized in a surgical setting [8], their findings are not necessarily generalizable to other clinical settings, especially those characterized by different workflows and team dynamics. Obstetrics, in particular, represents a fast‐paced, high‐risk setting that requires continuous multidisciplinary interaction, real‐time communication, and seamless coordination among the healthcare team to ensure patient safety. Applying the One Safe Act framework to obstetrics is a valuable extension of prior work as it offers a unique opportunity to examine how staff collaborate and communicate in high‐stakes and time‐pressured environments.

This study aimed to extend the application of the One Safe Act tool to the obstetric setting, with the goal of developing a thematic framework to inform individual clinical practices and institutional patient safety initiatives.

## 2 METHODS

Similar to the work by Duffy et al., this study is grounded in situated learning theory, which suggests that individuals learn by engaging in activities alongside community members, within a broader sociocultural context [8, 10]. As in the perioperative environment explored by Duffy et al., the labor floor serves as a strong example of a community of practice, where multidisciplinary professionals share common behaviors, experiences, and practices [8]. This setting fosters context‐specific, informal, experimental, and opportunistic learning, as described by the situated learning theory [8, 10].

Thus, we adapted the approach described by Duffy et al [8]. We collected community input by distributing an online survey to obstetric staff at a multicenter tertiary care academic medical institution. Unlike the in‐person, facilitated discussion format used by Duffy et al., our survey was administered in a flexible, asynchronous format to accommodate the high‐acuity and unpredictable nature of obstetric care, as well as to facilitate participation across shifts and healthcare roles. The survey was first introduced at the Mount Sinai's Departmental Quality and Safety Summit in January 2024, which was attended by 94 obstetric staff from Mount Sinai Hospital, Mount Sinai West, and Mount Sinai South Nassau. The Summit was open to all staff and was completely voluntary. After a brief introduction, participants were given approximately 15 minutes to access the quick response code and complete the survey. To increase participation and diversity across obstetric healthcare roles, the survey was subsequently distributed directly to the obstetric staff on the labor floors at Mount Sinai Hospital (distributed from May to June on three separate occasions) and Mount Sinai West (June–July on two separate occasions). Participants were approached across shifts and professional roles to ensure responses reflected a broad range of disciplines and positions within the obstetric care teams. We recirculated the survey multiple times to ensure sufficient role‐based representation, and data collection concluded when the data suggested sampling saturation, consistent with qualitative research methodology. No systematic differences were expected between Summit and labor floor respondents, as the survey prompt, eligibility criteria, and voluntary participation were identical across settings. Our approach mirrors that used by Duffy et al., who employed a convenience sample of perioperative staff (140 of 657 eligible participants) [8, 11].

The survey featured a free‐text response format, encouraging participants to reflect on their daily proactive safety behaviors in response to the following prompt:

Every day, there are many people like you who are doing ‘the small things’ for the safety of our patients. Your ONE SAFE ACT not only improves the care you provide to our patients but also shows your commitment to doing what's right. Let's declare our ONE SAFE ACT together, highlighting our personal commitment to patient safety and to our shared community. My ONE SAFE ACT is…

In addition to this open‐ended item, participants were asked to indicate their healthcare role (e.g., nurse, attending physician, resident, etc.) and primary hospital affiliation. One response was collected per participant. Responses were anonymized and stored in REDCap, a secure, password‐protected database compliant with the Health Insurance Portability and Accountability Act (HIPAA).

All interdisciplinary obstetric staff members from three hospitals within the Mount Sinai System (Mount Sinai Hospital, Mount Sinai West, Mount Sinai South Nassau) were eligible for inclusion (about 300 individuals) and participation was voluntary.

To analyze the data, we first identified the number of distinct behaviors in each free‐text response. If a response described multiple behaviors, each was analyzed separately. We then employed a combined deductive and inductive thematic analysis approach [12]. We used a “start list” of codes from the themes identified in Duffy et al. [8] (Table S1). One researcher (K.B.G.) applied these codes in a first reading of the data. Another researcher (R.M.C.) independently applied the start list codes to the data and refined and expanded the list based on emerging patterns in the data. The research team met to review the final coding scheme and organize key themes, with a third researcher (P.S.B.) participating to resolve discrepant opinions on coding application.

Each behavior was assigned a primary theme based on the behavior most prominently emphasized in each participant's description. Secondary and tertiary themes were assigned when responses reflected additional distinct behaviors or when a single behavior spanned multiple thematic domains. All theme assignments were determined through consensus among a three‐member research team, as described above. We then tabulated frequencies and percentages to summarize participant roles on the obstetric team and the distribution of primary themes across reported One Safe Act behaviors. To explore how themes co‐occurred, we created a network map to visualize the interconnections among primary, secondary, and tertiary themes. All visualizations were generated using R (version 4.4.1) [13, 14, 15].

## 3 RESULTS

Among over 300 eligible participants, a total of 141 obstetric staff members participated in the study, each providing a unique response to the One Safe Act survey. A total of 65 responses were collected at the Departmental Quality and Safety Summit, while the remaining 76 were obtained from survey distribution to obstetric staff on the labor floor. Across distribution methods, participant's primary hospital affiliations were Mount Sinai Hospital (*n* = 97, 68.8%), Mount Sinai West (*n* = 37, 26.2%), and Mount Sinai South Nassau (*n* = 7, 5.0%). Obstetrics (OB) nurses comprised the largest respondent group (47.5%), followed by OB attendings (21.3%), OB residents (9.2%), fellows (5.0%), and OB physician assistants (5.0%). Midwives, OB anesthesiologists, pediatric providers, OB nurse practitioners, and coaches from the Mount Sinai Patient Experience Office each accounted for less than 5% of the sample (Table 1).

**TABLE 1:** Participant characteristics.

Role	Participants No. (%) *N* = 141
Obstetrics nurse	67 (47.5)
Obstetrics attending	30 (21.3)
Obstetrics resident	13 (9.2)
Obstetrics fellow	7 (5.0)
Obstetrics physician assistant	7 (5.0)
Obstetrics anesthesiologist	6 (4.3)
Midwife	5 (3.5)
Non‐clinician	3 (2.1)
Pediatric provider	2 (1.4)
Obstetrics nurse practitioner	1 (0.7)

The 141 survey participants described a total of 151 “safety acts.” Eight participants described two behaviors in their respective free‐text responses, and one participant described three.

We identified seven key themes that encompassed the safety acts:

1. *Patient communication, education, and engagement* (e.g., “listen[ing] to patient concerns”)
2. *Staff communication and team routines* (e.g., “rounding” and “huddles”)
3. *Protocol adherence* (e.g., “check[ing] patient identifiers and allergies,” “timeout[s] before procedures,” and “medication verification”)
4. *Personal or team readiness* (e.g., “good breakfast” and proactively “review[ing] patient charts”)
5. *Prepared environment, equipment, and resources* (e.g., “checking emergency equipment to ensure everything is working and ready for immediate use”)
6. *Positive team culture* (e.g., “thanking people” and “asking staff how they are”)
7. *Routine‐based clinical care and documentation* (e.g., “accurate documentation”)

Table 2 provides detailed descriptions and representative examples of reported One Safe Act behaviors associated with each theme.

**TABLE 2:** Proactive safety behavior thematic classification scheme with descriptions and representative One Safe Act examples.

Theme	Description	Examples of One Safe Act collected
Patient communication, education, and engagement	Actively engaging with patients through listening and communication to enhance understanding and promote involvement in their care	Attendings: “listen to patient concerns,” “asking every patient more than once if they have any questions after telling them about their treatment plans”
		Nurses: “anticipating patient needs before they ask,” “listening to my patient and advocating for [them]”
		Residents: “making them feel comfortable, to allow them to trust the team,” “prior to leaving a patients’ room, I will always ask if there are any questions they have that haven't been addressed”
Staff communication and team routines	Informal and formal communication between members of the healthcare team through meetings, rounding, huddling, and sign‐outs to ensure coordination, collaboration, and continuity of care	Attendings: “review my plan of care with nursing,” “I review cases and evaluate them through QI process then give individual feedback”
		Nurses: “constant rounding,” “Writing out our goals for the day…,” “huddle with the team regularly and communicate the details of patient care”
		Midwife: “ensure the OB team is aware of my patient and their labor progress”
		Residents: “pm rounding to eval[uate] a [patient post transfusion,” “ensuring that there is constant communication between all members of the team"
Protocol adherence	Systematic safety checks, such as ID verification, medication confirmations, and procedure timeouts to prevent errors	Attending: “verify patient name, DOB and confirm their purpose of being on the labor floor”
		Nurses: “check patient identifiers and allergies before providing care,” “timeout before procedures,” “…scan the ID for medications and blood products,” “medication verification”
		Anesthesiologist: “checking drugs before administering”
Personal or team readiness	Pre‐duty or on‐duty activities that prepare individuals and teams to optimize performance	Attendings: “good breakfast,” “review the patient chart every time before I actually meet them for their appointment that day”
		Nurses: “deep breaths,” “leave on time”
		Midwife: “ask myself ‘what's missing’”
Prepared environment, equipment, and resources	Ensuring the availability and functionality of equipment, supplies, and workspaces for safe patient care	Resident: “I make sure the SCD machine is plugged in for future use”
		Nurses: “checking emergency equipment to ensure everything is working and ready for immediate use,” “to keep the entire team safe, I standardize the way we set up our instruments table so there are no variations in practice”
		Anesthesiologist: “ensuring operating rooms are set up for emergency cases”
Routine‐based clinical care and documentation	Consistent execution of clinical protocols and accurate documentation to maintain patient safety	Attending: “accurate documentation"
		Physician Associate: “making sure the dating of the patient is correct”
		Fellow: “I always clamp the Foley to the patients’ leg to avoid accidental urethral injuries”
Positive team culture	Fostering a respectful, collaborative, and supportive work environment to enhance teamwork and morale	Attendings: “[the] key is to introduce myself to the others in team (students, PA, CNMs, etc.) …,” “meet my nurses,” “asking staff how they are”
		Residents: “thanking people (specifically nurses) …,” “say hello and chat with the nursing staff at the beginning of the shift,” “starting the day with laughs…”

Abbreviations: CNM, certified nurse‐midwife; DOB, date of birth; ID, identification; OB, obstetrics; PA, physician assistant; QI, quality improvement; SCD, sequential compression device.

**FIGURE 1:**
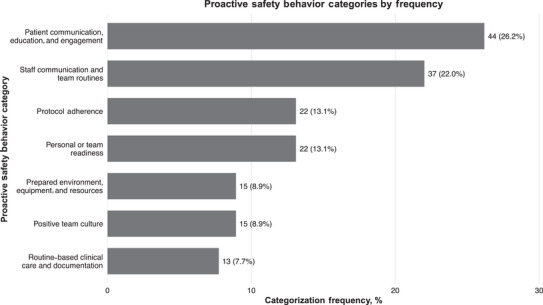
Frequency of proactive behavioral categories by primary assigned theme. *N* = 151 total behaviors identified from 141 total participants.

As shown in Figure 1, *Patient communication, education, and engagement* was the most assigned primary theme (44 behaviors, 26.2% of responses) followed by *Staff communication and team routine* (37 behaviors, 22.0%). *Protocol adherence* and *Personal or team readiness* were each assigned to 22 behaviors (13.1%). The remaining themes were each assigned to less than 10% of the total behaviors.

**FIGURE 2:**
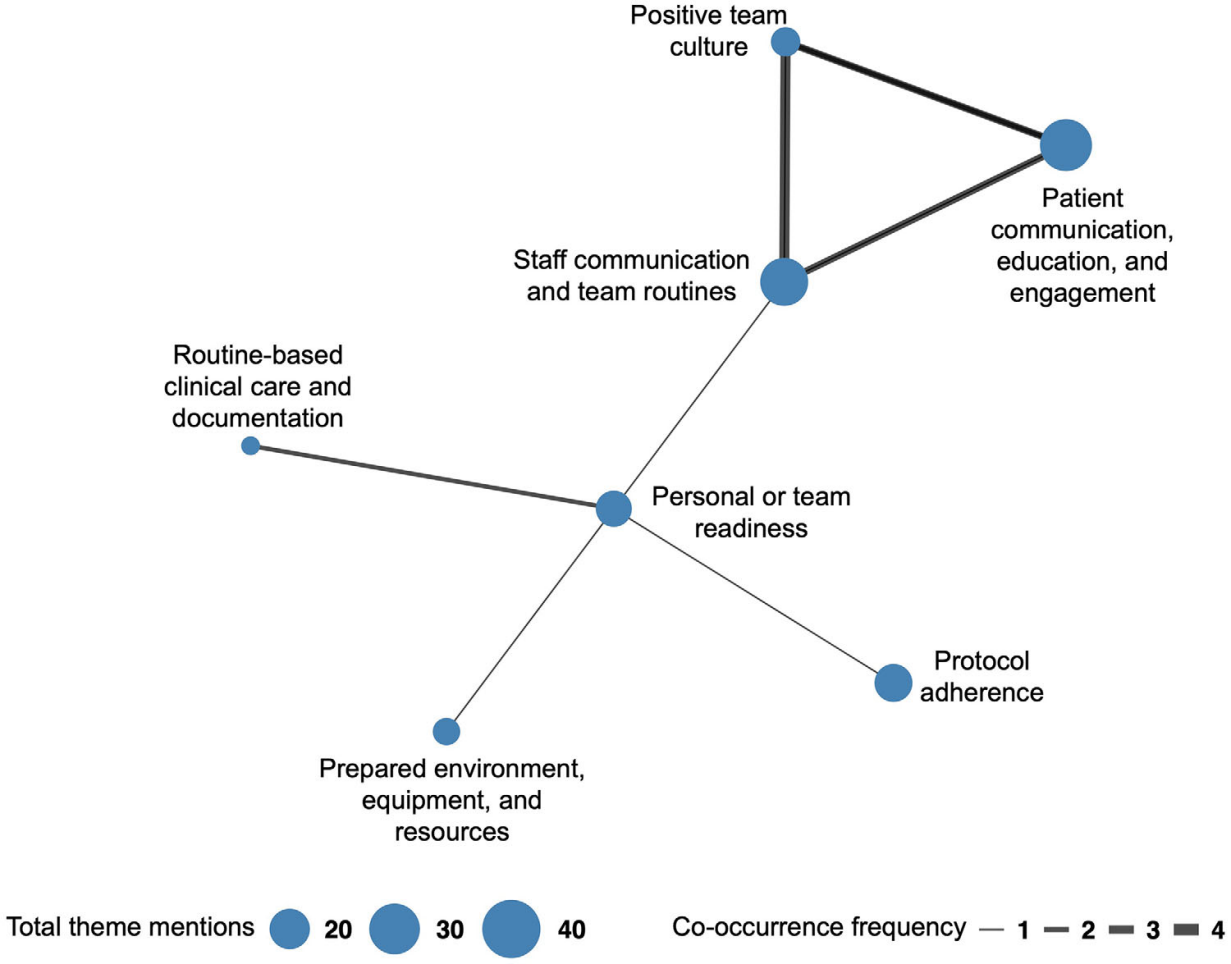
Network graph visualizing the overlap of proactive safety behavior themes. Each node (blue circle) represents a proactive safety behavior theme, with node size corresponding to the total number of times that theme was mentioned. Thirteen behaviors were assigned multiple themes (10 behaviors were assigned two themes, and three behaviors were assigned three themes). Edges (connecting lines) represent co‐occurrences between themes assigned to the same behavior. Thicker edges indicate more frequent co‐occurrence.

Thematic analysis revealed that some proactive safety behaviors spanned multiple themes, as illustrated in Figure 2. Of the 151 total behaviors, 10 were assigned a secondary theme, while three behaviors had both a secondary and a tertiary theme. Among these, *Patient communication, education, and engagement*; *Staff communication and team routines*; and *Positive team culture* were the most frequently overlapping themes (Figure 2).

## 4 DISCUSSION

We applied a rapid survey [8] in an obstetric hospital setting to identify proactive safety behaviors among interdisciplinary staff. We identified seven key themes describing individual behaviors to promote safety and quality, with the most frequently reported themes addressing *Staff communication and team routines* and *Patient communication, education, and engagement*.

Comparison of our findings with themes from the perioperative environment demonstrates the application and relevance of the One Safe Act tool across healthcare settings. Similar to Duffy et al. [8], we found a relatively high volume of routine‐based safety behaviors and attention to personal or team readiness to deliver safe care. Other prominent perioperative themes related to the availability of surgical supplies and ergonomic adaptations for lengthy procedures [8] appeared less frequently in our data, while communication (both provider–provider and provider–patient) emerged as central safety practices. Our findings align with key themes of communication, preparation, standardization, and teamwork highlighted in prior obstetric quality improvement research [16, 17, 18] and expand this research base by providing specific, first‐hand examples of how frontline obstetric staff actively implement these principles in daily practice.

*Patient communication, education, and engagement* was the most frequent theme in our data, supporting recommendations to enhance provider–patient communication to improve quality [19, 20]. Recent programs such as TeamBirth have achieved improvements in patient communication by implementing “team huddles” that include the patient and use a patient‐facing planning board [21]. These huddles have been associated with increased shared decision making [21], which is an important but underutilized component of patient engagement and may improve quality and safety in perinatal care [22, 23].

*Staff communication and team routines* was the second most common theme and is prominent in obstetric safety research [24]. For example, prior work identified structured tools such as huddles and team‐based rounds as promising and feasible interventions to improve communication and safety in perinatal care; both behaviors were frequently reported as One Safe Acts in our study [16, 22]. Additionally, in focus groups with labor and delivery staff, Grobman et al. found that effective coordination with external departments was the most cited communication barrier, followed by intradepartmental communication among obstetric staff [25]. None of our participants mentioned inter‐departmental communication, which may reflect the relative feasibility of implementing individual‐level safety acts or focusing on one's own unit or care team versus navigating inter‐departmental change. This aligns with the intent of the survey preamble, which emphasized “the small things” that staff do for patient safety daily, thereby encouraging respondents to highlight practical, immediately applicable behaviors rather than system‐level processes.

Prior studies have emphasized the importance of positive workplace culture and teamwork in patient safety [24, 25, 26]. In a qualitative positive deviance study of NYC hospital staff, positive nurse‐physician relationships distinguished hospitals with low rates of severe maternal morbidity from those with poorer performance [24]. In high‐performing hospitals, nurses felt valued and respected, while those in low‐performing hospitals reported feeling disrespected and blamed for adverse outcomes. Our participants identified meaningful actions that foster a positive culture, such as “asking staff how they are” or “greeting teammates at the start of a shift.” Additionally, we observed overlap between *Positive team culture*; *Staff communication and team routines*; and *Patient communication, education, and engagement*, suggesting that improvements in one area may simultaneously benefit other aspects of patient care.

By applying the Safety‐II paradigm, our study offers insights into what staff believe is already working on labor and delivery units at a large tertiary care center. These perspectives on “what goes right” can be complementary to Safety‐I paradigm research in identifying quality gaps and potential practical strategies for improvement. We demonstrate the use of the low‐resource “One Safe Act” exercise to center staff members' voices on their “personal commitment to patient safety and to our shared community.” Traditional safety interventions often require increasing staffing or responsibilities for existing employees in resource‐constrained obstetric units. Many of the “safety acts” identified in this study—such as “thanking people” and “listening to patient concerns”—are simple, actionable, resource‐neutral practices that individuals can adopt without additional financial or personnel investment. Although identifying these behaviors alone may not be sufficient to drive institutional change or improve outcomes, our study suggests a feasible first step in aligning teams around shared commitment to safe, high‐quality obstetric care. Future research should examine the impact of on staff awareness and engagement in quality improvement activities, as well as how the tool may be implemented alongside other interventions for multi‐level buy‐in and practice improvement.

This study has several limitations. First, the generalizability of results may be limited in settings with different institutional cultures. While including participants from three obstetric units strengthens the applicability of our findings across institutions, representation from Mount Sinai South Nassau was limited to Summit attendees, and unit‐specific cultures may not be fully captured. Additionally, the study relied on self‐reported behaviors and participants may overreport behaviors that align with institutional expectations or professional norms. Using a convenience sample may have also introduced bias by capturing individuals who are more available or inclined to participate compared to non‐responders. Furthermore, participants were recruited through both a departmental Summit (completely voluntary, open to all staff) and labor floor distribution. While we cannot exclude a potential difference between respondents recruited at the Summit and those recruited on the labor floor, we do not believe the groups are significantly different. Although the respondents represented diverse roles and provided cross‐disciplinary examples of behaviors, the results should be viewed as illustrative rather than fully representative of all staff. Moreover, we did not collect additional demographic information and therefore are unable to assess demographic diversity among respondents. Because our goal was to capture a broad spectrum of proactive safety behaviors rather than to highlight subgroup differences, we did not conduct a role‐ or institution‐specific thematic analysis.

Lastly, the One Safe Act tool is best utilized as a data collection and hypothesis‐generation tool. While participation in the One Safe Act activity may prompt individual or group reflection on proactive safety behaviors, this study was not designed to assess changes in awareness, comfort, or behavior, nor to evaluate the impact of reported behaviors on patient safety outcomes. Future work should evaluate methods to provide systematic feedback to teams and examine associations between reported behaviors and patient or process outcomes.

## 5 CONCLUSION

We applied the One Safe Act tool to the obstetric setting and identified seven themes of proactive safety behaviors, most prominently communication, protocol adherence, and readiness. By capturing real‐world safety practices at our institution, our findings bridge the gap between evidence‐based recommendations and actual everyday behaviors implemented by frontline healthcare professionals. While the One Safe Act tool is not a substitute for the commonly used and validated Safety‐I framework, it offers a complementary, low‐resource, simple approach to capture staff perspectives and focus on positive behaviors within a community of practice. Beyond simply data collection, the One Safe Act framework may serve as a foundation for reflection, team‐based discussion, and targeted quality improvement efforts. Future research should evaluate process‐level outcomes, such as staff engagement and positive workspace culture, as well as explore how using the One Safe Act framework may inform broader quality improvement initiatives.

## CONFLICT OF INTEREST STATEMENT

The authors declare no conflicts of interest.

## FUNDING INFORMATION

The authors received no specific funding for this work.

## ETHICS STATEMENT

The Icahn School of Medicine at Mount Sinai's Program for the Protection of Human Subjects determined that this study was exempt from human subjects research as defined by 45 CFR 46.104 (d).

## Supporting information



Supporting Information

## Data Availability

The data that support the findings of this study are available from the corresponding author upon reasonable request.
