# Supplementary material for: An application of the One Safe Act framework: Proactive safety behaviors in obstetric care
Source: Pregnancy (Hoboken). 2026 Mar 16;2(2):e70271. doi: 10.1002/pmf2.70271 (PMC13344204; doi:10.1002/pmf2.70271)
Supplement: Supplementary file 1 [file PMF2-2-e70271-s001.docx]

**Supplementary Table 1. Duffy et al. Proactive Safety Behavior Thematic Classification Scheme**

| **Name** | **Description** |
| --- | --- |
| **Routine-based adaptation** | Ensuring a routine, repetitive, highly practiced task relating to procedure, training, or proficiency is completed correctly. Failure to do this task (eg, checklist completion, checking allergies, using a certain piece of equipment every time a specific process is performed) could result in an unsafe situation |
| **Resource availability and assessment adaptation** | Reviewing and confirming that all required resources (including equipment, controls, and staffing) are present and functioning correctly (eg, checking that an ultrasound machine is available, ensuring that additional team members are available to assist). This can be an ongoing process |
| **Education adaptation** | Providing staff with new information that provides them with the knowledge to perform a task in a standard or safe way (eg, new defibrillator training) |
| **Environmental ergonomics adaptation** | Ensuring that the environment and equipment within it are configured appropriately for user interaction (eg, placing mats on floor to cover cable, ensuring that machines are adjusted to the correct settings). This can be an ongoing process |
| **Situational awareness adaptation** | Assessing situations for potential errors that could arise, adjusting behavior to defend against potential errors from occurring (eg, holding the door open for staff pushing a stretcher, recognizing a risk posed by beds being left unlocked, ensuring no equipment is hanging off the side of the bed while in transport, ensuring anesthetized patient safety) |
| **Communication and coordination adaptation** | Refers to the use of communication, coordination, teamwork, and planning to prevent errors and enhance safety. This includes provider-provider interactions (eg, using closed loop communication, practicing read-back, calling for help, assessing staff mix when assigning roles) and patient-provider interactions (eg, confirming laterality or procedure to be performed) |
| **Personal or team readiness adaptation** | Refers to pre-duty or on-duty activities or standard preparations required to perform optimally on the job such as adequate sleep (eg, review of appropriate medical/technical knowledge or patient-specific knowledge like chart or imaging review) |
| **Social awareness adaptation** | Attending to the social and emotional needs of others, including patients and junior colleagues (eg, putting patients at ease by showing empathy, helping patients feel more comfortable by offering to answer their questions) |

From Duffy C, Menon N, Horak D, et al. Clinicians' Perspectives on Proactive Patient Safety Behaviors in the Perioperative Environment. JAMA Netw Open. 2023;6(4):e237621. doi: 10.1001/jamanetworkopen.2023.7621
